# Case Report: Molecular Profiling Assists in the Diagnosis and Treatment of Cancer of Unknown Primary

**DOI:** 10.3389/fonc.2022.723140

**Published:** 2022-03-30

**Authors:** Bo Yu, Qifeng Wang, Xin Liu, Silong Hu, Liangping Zhou, Qinghua Xu, Yifeng Sun, Xichun Hu, Zhiguo Luo, Xiaowei Zhang

**Affiliations:** ^1^ Department of Medical Oncology, Fudan University Shanghai Cancer Center, Shanghai, China; ^2^ Department of Oncology, Shanghai Medical College, Fudan University, Shanghai, China; ^3^ Department of Pathology, Fudan University Shanghai Cancer Center, Shanghai, China; ^4^ Department of Nuclear Medicine, Fudan University Shanghai Cancer Center, Shanghai, China; ^5^ Department of Radiology, Fudan University Shanghai Cancer Center, Shanghai, China; ^6^ Canhelp Genomics Research Center, Canhelp Genomics, Hangzhou, China; ^7^ Institute of Machine Learning and Systems Biology, College of Electronics and Information Engineering, Tongji University, Shanghai, China

**Keywords:** gene expression profiling, next-generation sequencing, cancer of unknown primary, HER2 amplification, tumor of origin

## Abstract

**Background:**

For cancer of unknown primary (CUP), non-selective empiric chemotherapy is usually used. However, patients suffering from CUP are generally assumed to have a dismal prognosis with median overall survival of less than 1 year. Therefore, clinicians eagerly await the establishment of effective strategies for diagnosis and treatment. In recent years, the remarkable advances in next-generation sequencing (NGS) technology have enabled the wide usage of DNA/RNA sequencing to comprehensively analyze the molecular information of individual tumors and identify potential targets for patients’ diagnosis and treatment. Here, we describe a patient of CUP who was successfully diagnosed and treated with targeted therapy directed by comprehensive molecular profiling.

**Case Presentation:**

A 61-year-old Asian woman with a painless, slow-growing mass lesion in the mesosternum underwent fluorodeoxyglucose-positron emission tomography/computed tomography and was found to have malignant metastatic tumors in the mesosternum. Conventional pathological examination of metastatic lesions could not conclude the primary origin of the tumors. The patient was diagnosed with CUP at first. Then, comprehensive molecular profiling was employed to identify the tumor origin and genetic alterations. A gene expression-based tissue origin assay was performed using a tissue biopsy sample. The test result suggested that the lesion tumors might be breast cancer metastasis. Furthermore, liquid biopsy-based circulating tumor DNA profiling detected an *ERBB2* copy number amplification. Subsequent surgery and additional postoperative pathology analysis confirmed that the primary tumor site was indeed located in the right outer upper quadrant of the breast. After local surgical resection, the patient received 8 cycles of Docetaxel + Carboplatin + Trastuzumab + Pertuzumab (TCbHP) chemotherapy with subsequent *human epidermal growth factor receptor 2* (*HER2*)-targeted maintenance therapy. Currently, the patient is on regular follow-up and has achieved disease control for up to 6 months.

**Conclusion:**

Our findings suggest that molecular identification of the tumor origin and the detection of actionable molecular alterations may offer promise for improved diagnostic accuracy and important therapeutic implications for patients with the CUP syndrome.

## Introduction

Cancer of unknown primary (CUP) is a tumor that has metastasized from one part of the body to another part of the body. The place where it began, also called the primary site, is unknown. These cases make up about 3%–5% of all malignancies. The disease is characterized by late clinical presentation, early metastases, and poor prognosis, and the chance of 1-year survival is about 10%–20% (1). CUP often is challenging because it tends to be aggressive and has often spread to many parts of the body before it is found. CUPs are often found in the lymph nodes, liver, lung, peritoneum (lining of the bowel), or bone (2). In addition, because the origin of cancer is unknown, as once it has spread, it is difficult to locate its origin and so treatment and patient’s survival rate tend to be hindered. Thus, it may be more challenging to choose the best treatment (3).

With the advent of next-generation sequencing (NGS) techniques, massively parallel sequencing of tumor DNA offers great opportunity to identify actionable mutations and enables targeted therapy in oncologic practice (4–7). However, mutation profiling alone is not thought to be sufficient to guide the personalized treatment given that targeted therapies against a particular driver mutation can act differently in different tumor types. For example, BRAF V600E mutations are observed in cancers arising from numerous tissue sites, and the likelihood of response to BRAF inhibitors varies widely as a function of tumor type (8). Given the heterogeneous nature of the disease, the accurate diagnosis of primary tumor has an even greater role to optimize targeted therapy for patients with CUP.

Molecular tumor profiling has been under development over the last decade for predicting the tumor site of origins in patients with CUP (9, 10). Specific gene expression profiles have been well recognized in most cancers according to their site of origin, which reflects the different expression profiles present in their normal tissues of origin. Differences in gene expression thus allow distinction between various solid tumors and provide a valuable method for diagnosis of the tissue of origin in patients with CUP. Recently, a real-time PCR assay termed “the 90-gene expression assay” (Canhelp Genomics Co., Ltd., Hangzhou, China) was developed for the classification of 21 common tumor types based on gene expression profiling. Firstly, researchers established a pan-cancer transcriptome database with 5,434 specimens encompassing 21 tumor types to screen tumor-specific genes. Then, the Support Vector Machine Recursive Feature Elimination (SVM-RFE) algorithm was used to select the top 10 most predictive genes for each of the 21 tumor types. After removing redundant genes, a list of 90 genes was obtained and used to develop an SVM linear model named “90-gene classifier” for primary tumor identification. The 90-gene classifier was used to calculate the similarity scores of each tumor type, which reflect how much the gene expression pattern of the test sample is similar to the global gene expression pattern of a known tumor type. The similarity scores varied from 0 to 100, with the aggregate of all 21 similarity scores equaling 100. The tumor type with the highest similarity score was considered as the predicted tumor type by the 90-gene expression assay. In a retrospective cohort of 609 clinical samples, the gene expression assay demonstrated an overall accuracy of 90.4% for primary tumors and 89.2% for metastatic tumors. Furthermore, in a real-life cohort of 141 CUP patients, the gene expression assay was able to provide instructive predictions of primary tumors in 82.3% of patients (116/141). These findings suggest that the 90-gene expression assay could efficiently predict the primary origin for a broad spectrum of tumor types and support its diagnostic utility of molecular classification in difficult-to-diagnose metastatic cancer (11).

Here, we report a case of CUP that successfully achieved precise diagnosis and personalized treatment with targeted therapy of the disease based on comprehensive genomic analysis of the tumor and discuss the potential of using genomic tests to improve the diagnosis and management of CUP patients.

## Case Report

**Figure 1:**
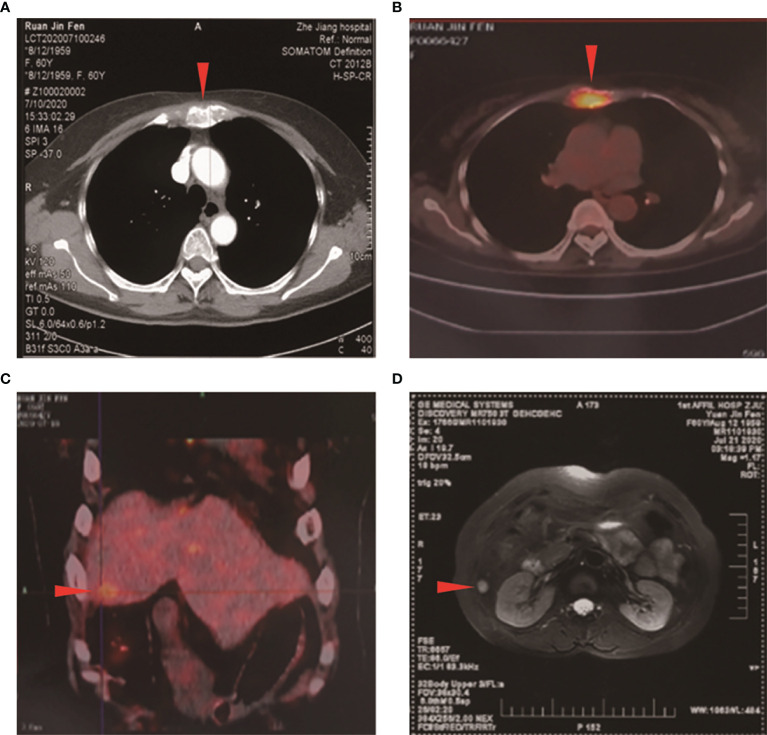
Initial computed tomography (CT), positron emission tomography/computed tomography, and magnetic resonance imaging (MRI) scans. **(A)** CT scan showed a space-occupying lesion in the mesosternum. PET-CT demonstrated high fluorodeoxyglucose (FDG) uptake in the **(B)** mesosternum and in the **(C)** right medial lobe of the liver (arrowhead). **(D)** MRI showed focal signal abnormalities in hepatic segment 8 (arrowhead).

A 61-year-old Asian woman, previously healthy, presented with a painless slow-growing mass lesion in the mesosternum. Her medical and family histories were unremarkable. Physical examination revealed no generalized lymphadenopathy. Chest computed tomography (CT) scan revealed space-occupying lesions in the mesosternum, a greater likelihood of malignancy, and multiple rounds or round-like nodules with variable sizes scattered throughout both lungs. Fluorodeoxyglucose (FDG)-positron emission tomography/computed tomography (PET-CT) detected two areas of bone destruction with soft tissue density shadow formation in the mesosternum with FDG uptake, malignant lesions, and possible metastases. Besides, low-density lesions in the right medial lobe of the liver showed poorly defined boundaries and slightly increased FDG metabolism; malignant lesion metastases were also considered. Bilateral thyroid gland showed heterogeneous density, a slightly low-density nodule in the right lobe, and poorly defined boundaries and slightly increased FDG metabolism; the middle segment wall of the esophagus was slightly thickened with slightly increased FDG metabolism, thus a combined gastroscopy test was recommended. Further liver magnetic resonance imaging (MRI) showed focal signal abnormalities in hepatic segment 8, inflammatory lesions were considered and malignant lesions were to be excluded, and liver puncture should be checked if necessary, also multiple hepatic hemangiomas (**Figure 1**). The thyroid ultrasound test revealed bilateral thyroid lesions with formation of multiple nodules (Thyroid Imaging Reporting and Data System, TI-RADS). The gastroscopic examination showed non-atrophic gastritis with erosion, fundic gland polyps (the greater curvature), HP (-), and chronic inflammation of antrum mucosa (the lesser curvature). The colonoscopy showed that the surface of the colorectal mucosa was smooth, and no significant abnormality was observed. However, these imaging evaluations plus endoscopy could not identify the primary location of the tumor. Significant laboratory investigations showed elevated tumor markers [Carbohydrate antigen19-9 (CA19-9): 38.80 U/ml↑, CA50: 32.02 IU/ml↑, Carcinoembryonic antigen (CEA): 10.00 ng/ml↑, Cytokeratin fragment antigen 21-1 (CYFRA21-1): 4.08 ng/ml↑]. The endoscopic ultrasonography showed multiple nodules in the liver (etiology to be determined), no obvious endobronchial space-occupying lesions were found in the pancreatic parenchyma, and no obvious abnormalities were observed in the gallbladder. Pathological examination of tissue from an ultrasound-guided fine-needle aspiration biopsy of the mesosternum lesions suggested a poorly differentiated metastatic carcinoma, with a bias toward classical adenocarcinoma. Immunohistochemical (IHC) detection failed to determine the exact tissue origin. The mammography detection showed that there were focal calcifications in the upper outer quadrant of the breast. Ultrasound examinations revealed that the bilateral breast had lobular hyperplasia, with formation of several nodules Breast Imaging Reporting and Data System (BI-RADS) in the upper outer quadrant of the breast, and no axillary lymphadenopathy was detected. In addition, MRI revealed that the upper outer quadrant of the breast exhibited focal mass with non-mass-like enhancement (BI-RADS 3). There was sternal metastasis with adjacent soft tissue thickening. Because the primary site could not be identified, we decided to treat the patient with local radiation therapy (6MV-X, 50GY/10) to the metastatic lesion of the sternum first.

**Figure 2:**
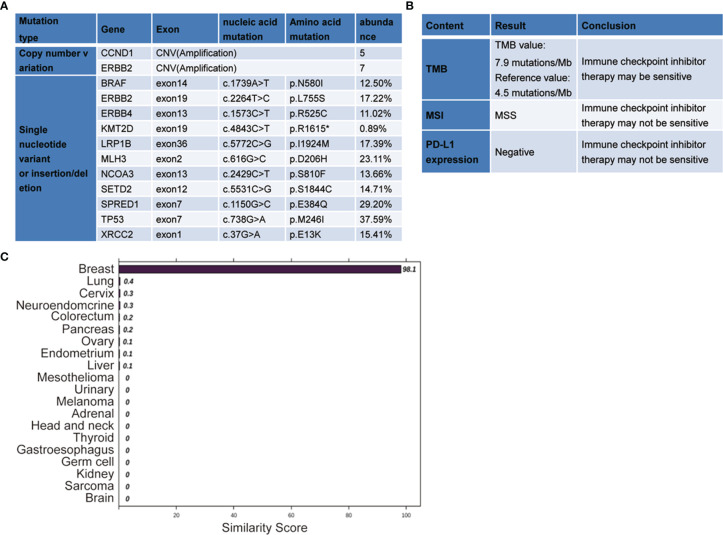
The next-generation sequencing (NGS)-based multiplex assay and the 90-gene expression assay results. **(A, B)** The gene expression profiling was analyzed by the NGS-based multiplex assay. **(C)** The 90-gene expression assay result with one similarity score for each of the 20 tumor types based on the formalin-fixed, paraffin-embedded tissues collected from the lesion in the mesosternum. The top three predictions were breast (98.1), lung (0.4), and cervix (0.3). Therefore, the most likely site is breast (98.1).

Comprehensive genomic analysis using the multiple-genes panel NGS that was designed to detect variants of cancer-related genes was performed. The genomic DNA was extracted from patient’s peripheral blood. NGS was performed to an average depth of >5,000x on the Novaseq 6000 platform (Illumina, USA). This assay demonstrated the presence of an *ERBB2* copy number variation (CNV), amplification. This amplification is well known to be a positive biomarker for trastuzumab, a recombinant humanized monoclonal antibody that specifically targets the extracellular site of *human epidermal growth factor receptor 2* (*HER2*). The genomic analysis also identified additional *CCND1* amplification and multiple gene mutations, including *TP53* (37.59%) mutation, c.738G>A, p.M246I; *SPRED1* (29.20%) mutation, c.1150G>C, p.E384Q; *MLH3* (23.11%) mutation, c.616G>C, p.D206H; *LRP1B* (17.39%) mutation, c.5772C>G, p.I1924M; *ERBB2* (17.22%) mutation, c.2264T>C, p.L755S; *XRCC2* (15.41%) mutation, c.37G>A, p.E13K; *SETD2* (14.71%) mutation, c.5531C>G, p.S1844C; *NCOA3* (13.66%) mutation, c.2429C>T, p.S810F; *BRAF* (15.50%) mutation, c.1739A>T, p.N580I; *ERBB4* (11.02%) mutation, c.1573C>T, p.R525C; and *KMT2D* (0.89%) mutation, c.4843C>T, p.R1615. Tumor mutational burden (TMB): 7.9 mutations/Mb. Microsatellitestable (MSS), Programmed cell death-Ligand 1 (PD-L1) (-) (**Figures 2A, B** **)**.

**Figure 3:**
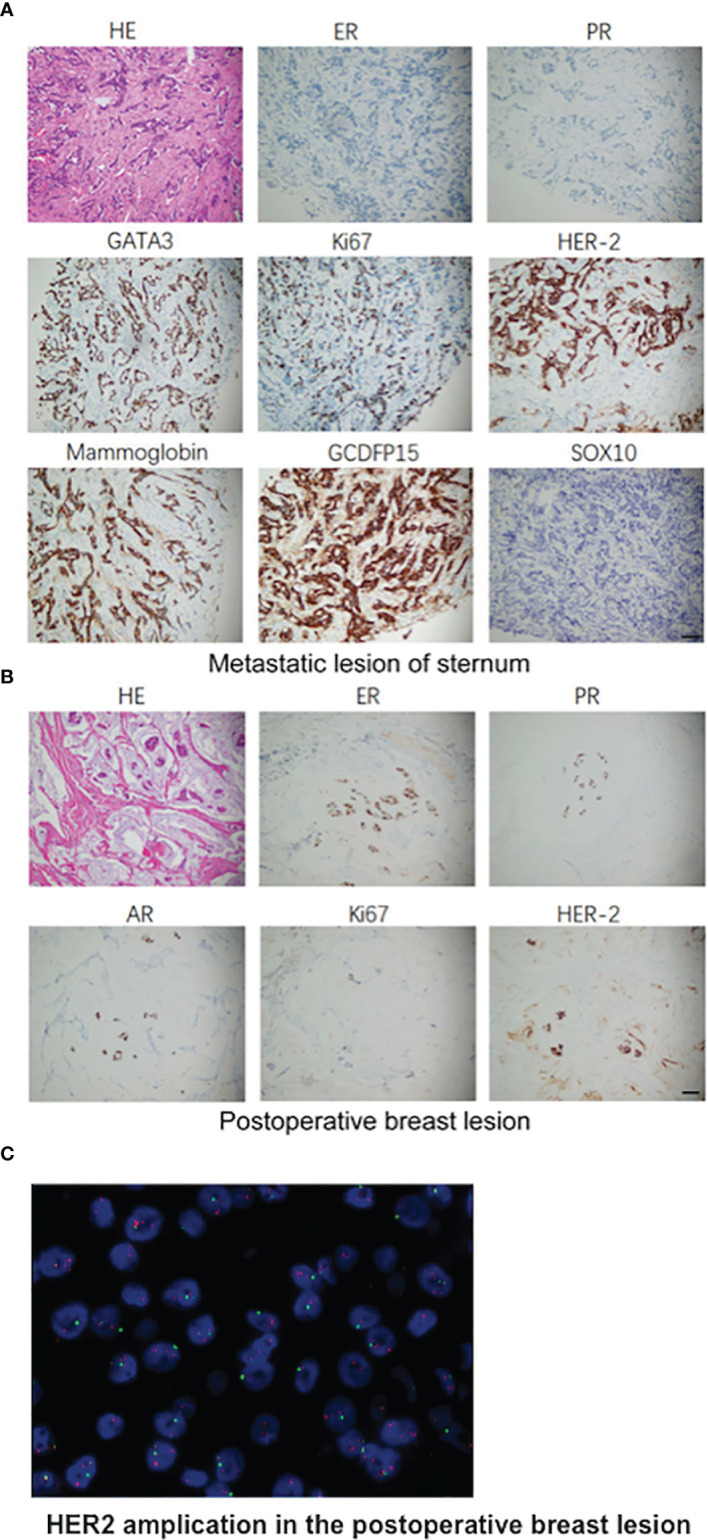
Pathologic findings. **(A)** Histopathological examination of the mesosternum lesions and the immunohistochemical examinations of the cells stained for ER (-), PR (-), HER2 (3+), Ki-67 (+50%), Mammaglobin (+), GATA3 (+), GCDFP15 (+), SOX10 (-). **(B)** H&E staining of the surgical specimens of total regional adenomammectomy and the immunohistochemical examinations of the cells stained positive for ER (+, 90%), PR (+, 70%), HER2 (2+), Ki-67 (+, 10%), and AR (+, 10%). **(C)** Representative image of HER2 amplification in the postoperative breast lesion using FISH analysis. Scale bar, 50 μm.

**Figure 4:**
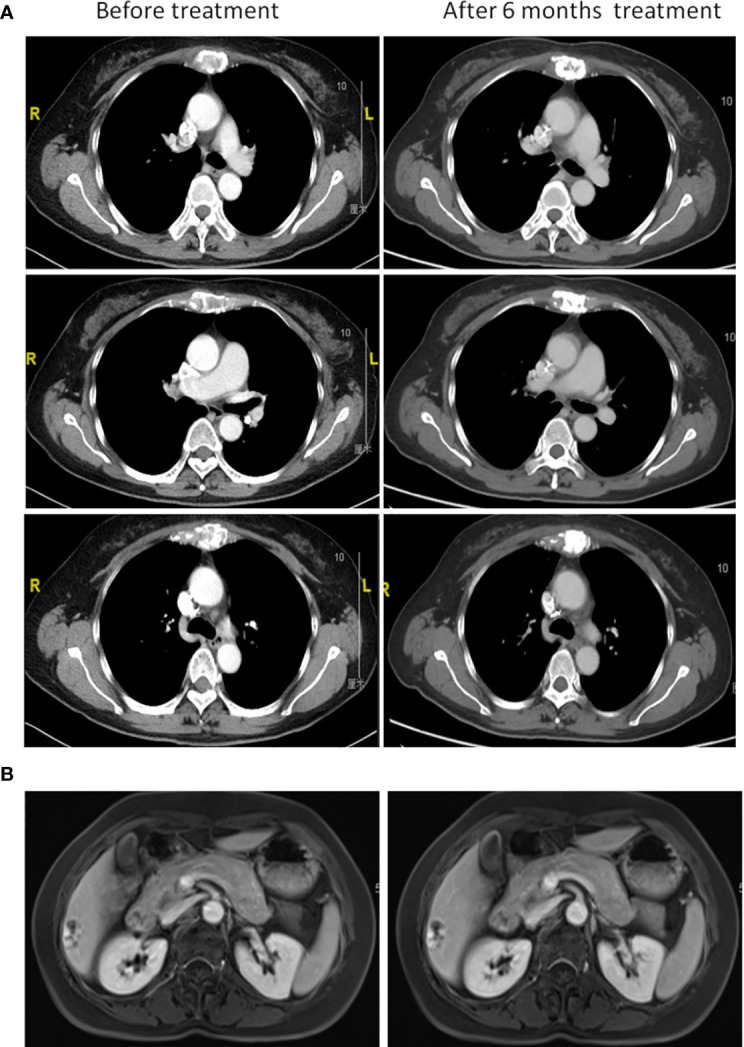
Clinical course after initiation of standard chemotherapy. **(A)** Chest computed tomography and **(B)** liver magnetic resonance imaging before chemotherapy treatment and after 6 months.

Furthermore, a 90-gene expression assay for identifying tumor tissue origin was performed based on a formalin-fixed paraffin-embedded (FFPE) biopsy sample of the patient’s mesosternum lesions. As shown in **Figure 2C**, these test results strongly supported a breast cancer as the cell of origin with a score of 98.1. Then, the patient underwent careful examination after the tissue-of-origin test with attention paid to the breast region. Histopathological examination of the mesosternum lesions revealed a poorly differentiated metastatic adenocarcinoma; combined with immunohistochemical findings, the cells stained for Estrogen receptor (ER) (-), Progesterone receptor (PR) (-), HER2 (3+), Ki-67 (+50%), Mammaglobin (+), GATA3 (+), GCDFP15 (+), and SOX10 (-) and tend to be breast cancer metastasis (**Figure 3A**). These findings suggested adenocarcinoma of breast origin. MRI and mammography-based tumor localization were subsequently utilized to perform the calcification focal resection in the right outer upper quadrant of the breast. Postoperative pathology confirmed it as invasive ductal carcinoma of the right breast, grade II with mucin secretion, and foci of invasive papillary carcinoma, with 0.7 cm in maximum dimension. Immunohistochemical analysis indicated that the cells stained for ER (+, 90%), PR (+, 70%), HER2 (2+), Ki-67 (+, 10%), and Androgen receptor (AR) (+, 10%) (**Figure 3B**). Fluorescence in situ hybridisation (FISH) detection revealed HER2 gene amplification (**Figure 3C**). After a local surgical resection, the patient has received monthly zoledronic acid treatment and 8 cycles of TCbHP regimen (Docetaxel + Carboplatin + Herceptin + Pertuzumab), with subsequent HP (Herceptin + Pertuzumab) maintenance therapy intravenously at a standard dose every 3 weeks for up to 6 months. These regimens were well-tolerated. The patient was regularly reviewed at monthly intervals. Six months after the operation, she was found to have stable disease, with the obvious osteogenic changes in metastatic lesions of the sternum (**Figure 4A**), and no change in the solitary liver lesion, which further confirmed that atypical hemangioma may be involved (**Figure 4B**). Tumor markers (before treatment: CA19-9: 38.80 U/ml↑, CA50: 32.02 IU/ml↑, CEA: 10.00 ng/ml↑, CYFRA21-1: 4.08 ng/ml↑) all decreased to normal levels.

## Discussion

CUP is defined as metastatic cancer in the absence of a clinically detectable anatomically defined primary tumor site after an adequate diagnostic evaluation. It constitutes 3%–5% of all newly diagnosed cancers per year worldwide. Including cancers of uncertain primary origin, the total number increases to 12%–15% of all newly diagnosed malignancies (12, 13). In the past decades, CUP patients commonly have poor prognoses due to treatment with a non-selective empirical therapy using cytotoxic agents (2). The era of precision medicine in oncology offers promise for improved diagnosis and better therapy for patients with the CUP syndrome. Gene expression-based tissue-of-origin molecular profiling has played an important role in the diagnostic armamentarium of CUP cancers. Besides, genomic characterization of CUP cancers using NGS techniques may also reveal actionable biomarkers for targeted therapies. Recently, research (14) has conducted a phase 2 clinical trial to evaluate the clinical benefit of comprehensive genomic profiling for CUP patients. Their findings demonstrate that site-specific treatment including molecularly targeted therapy based on profiling gene expression and gene alterations indeed contributes to treating patients with CUP.

Here, we present a patient who suffered from tumors with metastasis to the mesosternum, including a high likelihood of developing metastases in the liver, which usually indicates a poor prognosis (15). Her surgical specimen from mesosternum lesions first underwent conventional pathologic evaluations, which failed to accurately identify the primary site. Thus, we performed a novel 90-gene expression test based on an FFPE biopsy sample of the patient’s mesosternum lesions. The test results strongly suggested that the tissue of origin is breast cancer. Interestingly, the patient then underwent careful examination after the tissue-of-origin test with attention paid to the breast region. The MRI and mammography-based tumor localization indeed identified the primary tumor in the right breast. In the present case, considering that the remaining tissue biopsy sample was limited, the liquid biopsy-based circulating tumor DNA (ctDNA) profiling was performed afterward. The ctDNA profiling successfully identified oncogenic amplifications (*ERBB2, CCND1*) and mutations (*TP53, BRAF, ERBB2, ERBB4, *etc.). The *ERBB2* amplification has been widely reported as a driver CNV, which is most commonly observed in breast cancer (16) and is relatively fewer in gastric cancer, colorectal cancer, etc. (17–19). In breast cancer, the *ERBB2* amplification has been reported as a strong predictor of efficacy for trastuzumab that interferes with the *HER2*. Patients whose tumors harbor *ERBB2* amplification display a remarkable response rate in prospective trials of an anti-*HER2* monoclonal antibody, including randomized phase III trials (20). Accordingly, the patient received 8 cycles of TCbHP chemotherapy with subsequent *HER2*-targeted maintenance therapy. Despite the poor characteristics at baseline, the patient has survived to date with a high quality of life after the precise diagnosis and targeted therapy directed by comprehensive genomic profiling. Although advanced technology including the 90-gene expression assay and ctDNA profiling could help guide personalized therapy, it also has some limitations. Firstly, suboptimal specimens, including limited tissues, samples with excess necrosis, or little tumor contents, are not suitable for the 90-gene expression assay. Second, both methods, the 90-gene expression assay and the NGS tests, are not yet available in most resource-limited centers.

In summary, we present a patient who was first diagnosed as CUP was later successfully clarified as breast carcinomas and treated with targeted therapy based on transcriptomic classification and actionable mutation analysis. Our results suggest that molecular identification of the primary site and genetic mutations in tumors as a guide to selecting appropriate targeted therapy can result in a durable response for CUP patients. Further randomized studies comparing this systematic approach with empirical chemotherapy for CUP patients are warranted.

## Data Availability Statement

The original contributions presented in the study are included in the article/supplementary material. Further inquiries can be directed to the corresponding authors.

## Ethics Statement

The studies involving human participants were reviewed and approved by the Ethics Committee of Fudan University Shanghai Cancer Center. The patients/participants provided their written informed consent to participate in this study.

## Author Contributions

BY, QFW, and XWZ analyzed and interpreted the patient data and were major contributors in writing the article. XL, SLH, LPZ, XCH, QHX, and YFS contributed to the acquisition and analysis of data. XWZ and ZGL contributed to the concept and revised the article. All authors read and approved the final article.

## Funding

This work was financially supported by the Natural Science Foundation of China (Grant No. 81871948 and No. 81401986).

## Conflict of Interest

QHX and YFS were employed by the company Canhelp Genomics.

The remaining authors declare that the research was conducted in the absence of any commercial or financial relationships that could be construed as a potential conflict of interest.

## Publisher’s Note

All claims expressed in this article are solely those of the authors and do not necessarily represent those of their affiliated organizations, or those of the publisher, the editors and the reviewers. Any product that may be evaluated in this article, or claim that may be made by its manufacturer, is not guaranteed or endorsed by the publisher.

## Abbreviations

HER2, human epidermal growth factor receptor 2; CUP, cancer of unknown primary; FDG, fluorodeoxyglucose; NGS, next-generation sequencing; CT, computed tomography; MRI, magnetic resonance imaging; FFPE, formalin-fixed, paraffin-embedded; CNV, copy number variation; IHC, immunohistochemistry; TNBC, triple-negative breast cancer; TMB, tumor mutational burden.
